# Efficacy of Rituximab in CANOMAD: A Systematic Review

**DOI:** 10.7759/cureus.39237

**Published:** 2023-05-19

**Authors:** Alex S Aguirre, Ricardo A Vivanco, Juan Fernando Ortiz, Valery Rozen, Walter E InsuastI, John Fiallos, Camila Gallegos, Andrea Villavicencio, Kevin Salazar, Francisco Duenas, Ramit Singla

**Affiliations:** 1 School of Medicine, Universidad San Francisco de Quito, Quito, ECU; 2 Faculty of Health Sciences, Universidad Católica de Santiago de Guayaquil, Guayaquil, ECU; 3 Department of Neurology, Spectrum Health Medical Group, Grand Rapids, USA; 4 School of Medicine, Michigan State University College of Human Medicine, Grand Rapids, USA; 5 Division of Research and Academic Affairs, Larkin Health System, South Miami, USA; 6 Faculty of Health Sciences, Universidad de Guayaquil, Guayaquil, ECU; 7 Division of Research and Academic Affairs, Larkin Health System, Miami, USA; 8 Department of Vascular Neurology, University of Tennessee Health Science Centre, Memphis, USA

**Keywords:** opthalmoplegia, bulbar symptoms, igm, rituximab, gammapathy, canomad

## Abstract

CANOMAD, characterized by chronic ataxic neuropathy, ophthalmoplegia, immunoglobulin M (IgM) paraprotein, cold agglutinins, and disialosyl antibodies, encompasses a clinical, radiological, and laboratory diagnosis. CANOMAD is a rare condition, with fewer than 100 cases reported in the literature. The understanding and diagnosis of the disease have improved in the last few years, but the treatment of CANOMAD is mainly unknown, and there is not a clear consensus about it. We conducted a systematic review regarding the efficacy of rituximab in CANOMAD's treatment to investigate the clinical and biological response of CANOMAD in patients treated with rituximab. We used the Preferred Reporting Items for Systematic Reviews and Meta-Analyses (PRISMA) and Meta-Analyses of Observational Studies in Epidemiology (MOOSE) reporting guidelines for this systematic review. To analyze the bias of the study, we used the Joanna Briggs Institute's (JBI) Critical Appraisal Checklist to analyze the bias of the case reports, and we used the Risk of Bias in Non-Randomized Studies of Interventions (ROBINS-I) tool for the observational studies.

We only included case reports, case series, and observational studies written in English with patients formally diagnosed with CANOMAD and treated with rituximab. We excluded systematic reviews, literature reviews, and meta-analyses. We investigated the clinical and biological responses of the patients to rituximab. The clinical response was classified as complete recovery (CR), partial response (PR), stable disease (SD), and non-response (NR).

We gathered 34 patients. The literature uses a modified Rankin score to define complete improvement (CR), partial response (PR), stable disease (SD), and progression. Clinically, there were three patients with CR, five with PR, 15 with SD, and 11 with progression. The biological response was assessed by measuring the decrease in antibody titers in 27 patients. Among those, six patients had CR, 12 had PR, eight had SD, and one had progression. Among 15 patients with neurological evaluation, 10 had ocular symptoms, and two presented with bulbar symptoms. Seven of the ten patients with ocular symptoms had SD, two had PR, and one had progression. Only 14 patients had a report of demyelinating features. Three had an axonal pattern, six had a demyelinating pattern, and five had a mixed pattern. Among patients with an axonal pattern, three had an SD. Among patients with a demyelinating pattern, three had a PR, two had an SD, and one had progression. Among patients with a mixed pattern, four had SD, and one had progression.

We concluded that patients with CR have a shorter disease duration than patients with PR, SD, or progression. In addition, patients with CR had longer follow-ups than the other groups, suggesting that being treated early with rituximab improves the clinical outcome and has a sustained effect. There were no differences in the frequency of ocular and bulbar symptoms among patients with CANOMAD. The axonal pattern is more common in patients with SD, suggesting that axonal and mixed patterns could be markers of a bad prognosis.

## Introduction and background

CANOMAD (chronic ataxic neuropathy, ophthalmoplegia, immunoglobulin M (IgM) paraprotein, cold agglutinins, and the presence of disialosyl antibodies) is a rare type of gammopathy that includes a series of clinical, radiological, and laboratory features [1]. CANOMAD is a rare condition, with less than 100 cases reported in the literature [2]. The disease usually presents with peripheral neuropathy and chronic sensory ataxia. Pulmonary and ocular symptoms develop in the later stages of the disease as relapsing symptoms [2].

The features and progression of CANOMAD are usually variable [1]. The most common clinical features include sensory symptoms (78%), ataxia (47%), ophthalmoplegia (13%), bulbar symptoms (7%), facial nerve palsy (4%), motor weakness/myoclonus (7%), and dyspnea (2%) [1]. Importantly, the disease is usually associated with malignancy in 38% of the cases, with Waldenström macroglobulinemia being the most commonly associated malignancy [1]. The course of the disease can have a relapsing-remitting pattern (31%), be chronically progressive (67%), or present with symptomatic flare-ups (2%) [1].

Canoma is caused by IgM antibodies against disialosyl antibodies (GQ1b, GT1B, or GD1b) [3]. On a microscopic level, initially, there is an IgM antibody/antigen reaction against disialosyl epitopes at nodal and para-nodal regions [3]. Eventually, this response progresses from antigen-dependent B-cell proliferation to antigen-independent B-cell proliferation, activating the complement, which leads to membrane attack complex (MAC) activation, causing a disruption of sodium channels and causing structural lesions [3]. On a pathological level, the most commonly affected structures are nerves, nerve roots, nerve root ganglions, and the dorsal columns [4].

The diagnosis of the disease starts with the investigation of IgM peripheral neuropathy, as suggested by Le Cann et al. [1]. The process for this is: 1) perform nerve conduction studies to differentiate between axonal and demyelinating patterns; 2) detect the presence of anti-MAG antibodies (anti-myelin-associated glycoprotein); 3) order serum-free light chain concentration and ratio; 4) screen for red flag features such as dysautonomia, weight loss, cutaneous signs, and heart, kidney, or lung involvement to rule out cryoglobulinemia or amyloidosis; 5) confirm the diagnosis with GQ1b, GT1B, or GD1b antibodies [1].

The understanding and diagnosis of the disease have improved in the last few years, but the treatment of CANOMAD is mainly unknown, and there is not a clear consensus about it. The first line of treatment is intravenous immunoglobulin (IVIG). Steroids are not effective. We conducted a systematic review regarding the efficacy of rituximab, an anti-CD20 monoclonal antibody, in CANOMAD's patients to investigate the clinical and biological response of this disease to rituximab.

## Review

Methods

Protocol

To conduct this systematic review, we used the Preferred Reporting Items for Systematic Reviews and Meta-Analyses (PRISMA) protocol [5].

Eligibility Criteria and Study Selection

We included case reports and observational studies conducted on humans that were written in English and published after 1985. We included studies with the following characteristics: (1) population: patients diagnosed with CANOMAD; (2) intervention: patients treated with rituximab; (3) comparison: there is not a comparison group; (4) outcomes: clinical and biological response of rituximab in patients with CANOMAD or other chronic ataxic neuropathies with disialosyl antibodies (CANDA).

The clinical response was determined based on the criteria established by Le Cann et al. [1] and Garcia-Santibanez et al. [6], which focus on the improvement of neurological deficits and modified Rankin score (mRS). A complete clinical response (CR) was identified as the resolution of all symptoms or an mRS score of 0. A partial clinical response (PR) was defined as the resolution of some symptoms or a decrease of at least one point in the RS. Disease stabilization was determined by the absence of new deficits or relapses or no change in RS. Disease progression was characterized by new deficits, relapses, or an increase of one or more points in the RS. These criteria were used to assess the clinical response to treatment in the study.

The biological response was measured based on the changes in serum IgM antibody levels, similar to the study by Le Cann et al. [1]. This variable can be categorized into four groups: complete response (CR), partial response (PR), stable disease (SD), and progression. CR is characterized by the disappearance of IgM and negative immunofixation, indicating a complete resolution of the condition being treated. PR is defined as a decrease in more than 50% of serum IgM levels, indicating a significant improvement in the condition. SD refers to a minor change in serum IgM levels, either an increase of less than 25% or a decrease of more than 25%. Conversely, progression is indicated by an increase in serum IgM levels of more than 25%, suggesting a worsening of the underlying condition.

Data Extraction and Analysis

We collected the following information from each paper: author, year, clinical response, biological response, the pattern of nerve conduction studies (demyelinating, axonal, or mixed), presence of ocular or bulbar symptoms, disease duration, and follow-up time.

Database and Search Strategy

We used PubMed as the database with the following search terms: ("CANOMAD" (Title/Abstract) AND "RITUXIMAB" (Title/Abstract)) OR ("CANDA" (Title/Abstract) AND "RITUXIMAB" (Title/Abstract)) OR ("CANOMAD" (Title/Abstract) AND "TREATMENT" (Title/Abstract)). 

Bias Assessment

We applied the Joanna Briggs Institute's (JBI) Critical Appraisal Checklist for case reports to assess the risk of bias in the case report studies analyzed in this publication.

Results 

**Figure 1:**
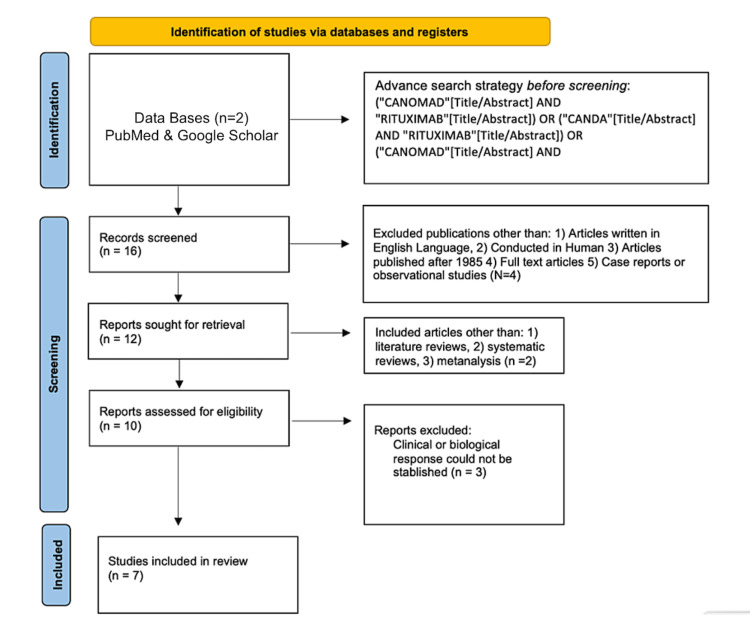
PRISMA flow chart of the systematic review

Figure 1 shows the results of the study using a Preferred Reporting Items for Systematic Reviews and Meta-Analyses (PRISMA) flow chart.

Study Outcomes 

Of 34 patients, rituximab's effect on clinical response was as follows: three patients showed a complete response, 11 had a partial response, 15 had stable disease, and five showed progression. Those with a complete response had an average duration of their disease of four years, with an extended follow-up of 10 years. Those with stable disease had the longest disease duration, at 8.5 years. Table 1 shows the outcomes of this systematic review [1, 6-11].

**Table 1:** Outcomes of the systematic review CR: complete response; PR: partial response; SD: stable disease

	N (%)	Mean disease duration	Mean follow-up duration
CR	3 (8%)	4 years	10 years
PR	11 (32%)	6 years	2 years
SD	15 (44%)	8.5 years	4 years
Progression	5 (15%)	5 years	2 years
Total	34		

Table 2 shows the frequency of biological and clinical responses among patients using rituximab [1, 6-11]. 

**Table 2:** Frequency of biological and clinical response CR: complete response; PR: partial response; SD: stable disease; NR: no response

Biological response	Frequency	Clinical response	Frequency
CR	6	CR	3
PR	12	PR	11
SD	8	SD	15
NR	1	NR	5
Total	27		34

The biological response was evaluated in 27 patients based on the decrease in antibody titers. Among them, six patients showed a complete response, 12 had a partial response, eight had stable disease, and only one had progression. There was no visible association between the clinical and biological responses. Table 3 shows the biological response in these patients in the systematic review [1, 6-11].

**Table 3:** Outcomes of the systematic review CR: complete response; PR: partial response; SD: stable disease

		Clinical response				
		CR	PR	SD	Progression	Total
Biological response	CR	1	1	4	0	6
	PR	1	7	3	1	12
	SD	0	1	6	1	8
	Progression	1	0	0	0	1
	Total	3	9	13	2	27

Among 15 patients with neurological evaluation, 10 had ocular symptoms, and two presented with bulbar symptoms. Seven out of the 10 patients with ocular symptoms had disease stabilization; two responded partially, and one progressed. Patients with a complete clinical response did not present ocular or bulbar deficits. Table 4 shows the bulbar and ocular manifestations among the patients in this review [1, 6-11].

**Table 4:** Nerve conduction studies in patients with CANOMAD CR: complete response; PR: partial response; SD: stable disease

	Axonal	Demyelinating	Mixed	Total
CR	0	0	0	0
PR	0	3	0	3
SD	3	2	4	9
Progression	0	1	1	2
Total	3	6	5	14

Regarding the electrophysiologic pattern of CANOMAD, there was no association with the clinical response to rituximab therapy. Unfortunately, none of the patients with a complete response had information about their electrophysiologic studies. The three patients with partial clinical responses had a demyelinating pattern among the rest. On the other hand, among those with stable disease, three had an axonal pattern, two had a demyelinating pattern, and four had a mixed pattern. Finally, those two patients without clinical response had demyelinating and mixed patterns. Table 5 shows the ocular and bulbar symptoms (frequency) in this systematic review [1, 6-11].

**Table 5:** Ocular and bulbar manifestations among patients with CANOMAD. Patients with complete responses did not present ocular or bulbar deficits. PR: partial response; SD: stable disease

	Ocular	Bulbar	Total
PR	2	1	4
SD	7	1	9
Progression	1	0	2
Total	10	2	15

Bias Analysis 

We applied the JBI Critical Appraisal Checklist for case reports to assess the risk of bias in the case report studies analyzed in this publication. Table 6 shows the case reports [7-11].

**Table 6:** Bias analysis of case reports and case series

					Risk of bias
	Selection (1*)	Ascertainment (max 2*)	Causality (max 4*)	Reporting (1*)	
Siddiqui et al, 2003 [10]	*	**	**	*	Low
Delmont et al, 2010 [8]	*	**	**	*	Low
Loscher et al, 2013 [9]	*	**	*	*	Moderate
Marastoni et al, 2020 [11]	*	*	*	*	High
Salamon et al, 2020 [7]	*	**	**	*	Low

We applied the Risk of Bias in Non-Randomized Studies of Interventions (ROBINS-I) criteria to evaluate the bias of the observational studies [12]. Table 7 shows the bias analysis of the observational studies [1,6]. 

**Table 7:** Bias analysis of the observational studies

Author, year	Confounding	Selection of participants	Classification	Deviations	Missing data	Measurements	Selection of reported results
Garcia-Santibanez et al, 2018 (3)	Low risk	Low risk	Medium risk	Low risk	Medium risk	Moderate risk	Low risk
Le Cann et al, 2020 (2)	Low risk	Low risk	Medium risk	Low risk	Medium risk	Medium risk	Medium risk

Discussion

Our systematic review confirms the results of previous observational studies and case reports, indicating that rituximab may be effective in controlling the progression of CANOMAD [1,6-10,13]. In our study, 41% of patients exhibited a clinical response (complete or partial), while two-thirds of patients had a biological response. However, our findings differed from those of Le Cann et al. [1] and Santibañez et al. [6], where the clinical response to rituximab was 52% and 27%, respectively, while the biological response was 57% and 77%, respectively. Notably, only 8.8% of patients had a complete response to rituximab, suggesting that the treatment may be less effective in modifying the natural course of CANOMAD. Interestingly, the biological response was superior to the clinical response in the three studies, indicating that other pathological mechanisms unrelated to antibodies or irreversible damage may explain the lack of correlation between the two responses.

Our evaluation of ocular and bulbar symptoms revealed no significant differences in the frequency of these symptoms among patients with CANOMAD and the use of rituximab. Most of the patients with ophthalmoplegia (70%) had disease stabilization, while patients with bulbar symptoms were less responsive to treatment. The non-improvement of non-peripheral manifestations with rituximab or other immunosuppressive agents suggests that these deficits may have non-antibody-related pathogenesis.

Regarding electrophysiologic findings, demyelinating and mixed patterns were the most common in CANOMAD. Half of the patients with demyelinating patterns had a partial response compared to patients with axonal and mixed patterns, who only achieved disease stabilization. This difference may be due to the potential for myelin sheath repair with appropriate treatment. However, caution must be exercised in generalizing these findings to all patients with CANOMAD.

CANOMAD is a chronic neuropathy with disialosyl antibodies, unlike Miller Fisher Syndrome (MFS), which is an acute neuropathy against similar disialosyl antibodies. Compared to CANOMAD, MFS is usually reversible despite having similar pathological pathways [14].

As with most neuropathies, CANOMAD affects the nerves and nerve roots, as there are circulating antibodies against the disialosyl epitopes in the nodal and paranodal areas of the nerves and nerve roots. In addition, the disease produces antibodies against the nodal and paranodal regions of the nerve [4]. Furthermore, reported biopsies in other patients with CANOMAD have reported dorsal column atrophy, which would explain the chronic ataxia in these patients [15]. In both conditions, the antibody reacts against the nodal area first, and then there is an extension to the paranodal area [4]. In the nodal area, the sodium channels reappeared on both sides of the node. In CANOMAD, the sodium channel and paranodal proteins such as Caspar and Constantin 2 eventually disappear, leading to axonal dysfunction [4].

Rituximab has proven to be effective in other monoclonal gammopathies by decreasing the antibodies causing the diseases, such as chronic inflammatory demyelinating polyradiculoneuropathy (CIDP), anti-MAG, or multifocal motor neuropathy (MMN) [16]. Moreover, rituximab has also been effective in treating autoimmune neuropathy [17]. However, the first time that rituximab showed to be effective was with a patient with CANOMAD [10]. Posteriorly, there have been clinical trials that showed that rituximab was effective in two clinical trials of patients with anti-MAG neuropathy and chronic inflammatory demyelinating polyneuropathy (CIDP), and there have been observational studies where rituximab has proven to be effective in paranodal autoimmune neuropathies [17-21]. 

In a systematic review, rituximab proved effective in 47% of patients with anti-MAG neuropathy, 63% with CIDP, and 96% with autoimmune neuropathy. Rituximab appears to reduce humoral response by binding to CD20 on the B cell surface, causing a depletion of B cell lymphocytes, which causes a reduction of antibody titers, cell-mediated immunity, and complement deposition [16]. Intravenous immune globulin (IVIG) may be used as first-line therapy for this syndrome, as it has proven to provide a response. Steroids, on the other hand, are ineffective for this disorder [1, 22].

There are several limitations to consider in this study, mostly related to the difficulties of studying rare diseases other than CANOMAD. First, this study was a retrospective analysis, and the data were collected from observational studies and case reports, which could have led to incomplete or inaccurate data. The sample size was also relatively small, which limits the statistical power of the study. Also, the study did not have a control group, making it difficult to determine whether the observed responses were due to the treatment or other factors such as natural disease progression or spontaneous remission. Finally, the study only evaluated the clinical and biological outcomes of rituximab treatment and did not assess the safety or tolerability of the treatment. Further studies are needed to evaluate the safety and long-term outcomes of rituximab treatment in patients with CANOMAD. It may also be useful to investigate the underlying mechanisms of the disease and identify potential therapeutic targets that could complement or enhance the effects of rituximab.

## Conclusions

The biological response is more significant than the clinical response in a patient with CANOMAD, as there is more pathological involvement beyond the nerve and the nerve root. Compared to MFS, CANOMAD is usually irreversible as permanent nodal and paranodal dysfunction exists.

Rituximab is a proven therapy in other gammopathies and was effective in the sample of patients we studied. In addition, patients with CR had longer follow-ups than the other groups, suggesting that being treated early with rituximab improves the clinical outcome and has a sustained effect. Bulbar symptoms were infrequent among our patients, while ocular symptoms were relatively common. The axonal pattern is more common in patients with SD, suggesting that axonal and mixed patterns could be markers of a bad prognosis.
